# A comparative study of robot-assisted thoracoscopic surgery and conventional approaches for short-term outcomes of anatomical segmentectomy

**DOI:** 10.1007/s11748-023-01983-y

**Published:** 2023-11-07

**Authors:** Tomohiro Haruki, Yasuaki Kubouchi, Yoshiteru Kidokoro, Shinji Matsui, Takashi Ohno, Shunsuke Kojima, Hiroshige Nakamura

**Affiliations:** Department of Surgery, Division of General Thoracic Surgery, Faculty of Medicine, Tottori University, 36-1 Nishi-cho, Yonago, Tottori 683-8504 Japan

**Keywords:** Lung cancer, Segmentectomy, Robot-assisted thoracoscopic surgery, Propensity-score matching

## Abstract

**Objectives:**

Since anatomical segmentectomy requires meticulous dissection of the segmental pulmonary vessels and bronchus, robot-assisted thoracoscopic surgery (RATS) has been widely adopted in recent years. We investigated the usefulness of RATS segmentectomy by comparing perioperative outcomes with conventional approaches including open thoracotomy or video-assisted thoracoscopic surgery (VATS). We compared perioperative outcomes of segmentectomy between RATS and conventional approaches including open thoracotomy or video-assisted thoracoscopic surgery (VATS).

**Methods:**

This single-institutional retrospective study comprised 231 patients with primary lung cancer who underwent segmentectomy by RATS or conventional approaches between January 2011 and December 2022. Surgical outcomes and postoperative complications were analyzed among patients whose background factors were adjusted by propensity score matching (PSM).

**Results:**

Before PSM, there were significant differences in age, smoking status, and types of segmentectomy. After PSM, 126 patients (63 patients in each group) were included in this analysis. The RATS group had significantly shorter operative time (154 vs 210 min; *p* < 0.01), fewer bleeding amounts (10 vs 40 mL; *p* < 0.01), and shorter duration of chest drainage (2 vs 2 days; *p* = 0.04) than the conventional-approach group. There was no significant difference in the incidence of all complications; however, the incidence of postoperative pneumonia was significantly lower than in the conventional-approach group (*p* = 0.02).

**Conclusions:**

RATS segmentectomy is proposed to be useful. It was suggested that RATS segmentectomy may be useful with better perioperative results than the conventional approach. Further studies on oncological long-term outcomes and cost–benefit comparisons are needed.

**Supplementary Information:**

The online version contains supplementary material available at 10.1007/s11748-023-01983-y.

## Introduction

For a long time, we have performed lobectomy for early-stage (T1N0) non-small cell lung cancer (NSCLC) based on the results of a landmark study published by the Lung Cancer Study Group in 1995 [1], which showed that sublobar resection was associated with an inferior overall survival and a threefold increase in the local recurrence rate compared with lobectomy. However, following the results of the Japan Clinical Oncology Group (JCOG) series (JCOG0802/WJOG4607L and JCOG1211) and Cancer and Leukemia Group B (CALGB) 140503 [2–4], both of which are pivotal clinical trials published recently, sublobar resection for small-sized, early-stage NSCLC could be considered an oncologically effective alternative to lobectomy. As long as sufficient surgical margins and negative lymph node metastasis are provided, anatomical segmentectomy is a beneficial procedure with the advantage of preserving respiratory function and the chance of a second lung cancer operation. This procedure will undoubtedly be required more in the near future because pulmonary nodules with ground-glass opacity have been frequently detected by virtue of computed tomography (CT) screening and other factors.

Open thoracotomy or video-assisted thoracoscopic surgery (VATS) has historically often been the standard approach chosen for segmentectomy; however, in recent years, robot-assisted thoracoscopic surgery (RATS) has been accepted as a less-invasive and advantageous approach for segmentectomy. The features of RATS, such as forceps manipulation with articulation and three-dimensional (3D) thoracoscopic field of view, can be utilized in segmentectomy, which requires more meticulous manipulation than lobectomy.

To date, few studies have compared RATS with open thoracotomy or VATS segmentectomy for primary lung cancer [5–9]. In this study, we investigated the usefulness of RATS segmentectomy by comparing perioperative outcomes with those of conventional approaches, including open thoracotomy or VATS, among lung cancer patients whose background factors were adjusted by propensity score matching (PSM).

## Patients and methods

### Patient selection

This study was approved by our facility’s institutional review board in November 2019 (19A143), which waived the patients’ written informed consent requirement because of the study’s retrospective design. We reviewed 231 consecutive patients with primary lung cancer patients who were treated by open thoracotomy or thoracoscopic (including VATS and RATS) anatomical segmentectomy and lymph node dissection at our institution from January 2011 to December 2022. Thoracoscopic surgery (RATS or VATS) were generally indicated for patients with clinical stage I–IIA disease; those with clinical stage IIB or higher were indicated for thoracoscopic surgery only when considered technically feasible. Patients who underwent induction therapy, incomplete resection microscopically or macroscopically (R1 or R2 resection), and conversion from segmentectomy to lobectomy were excluded from this study. All patients underwent preoperative contrast-enhanced CT of the chest and upper abdomen within 1 month before their surgery. Primary tumors were evaluated by chest CT, and their sizes were determined by thin-section CT findings. For all tumors, we obtained the tumor’s maximum dimension (tumor) and its solid component (consolidation) using a lung window-level setting from thin-section CT images, and then estimated the consolidation/tumor ratio (C/T ratio) for each tumor [10]. Pulmonary vessels and bronchi on CT were constructed on CT in 3D using Synapse Vincent (Fuji Film, Tokyo, Japan) to perform preoperative surgical simulation. Magnetic resonance imaging of the brain and positron emission tomography/CT were routinely performed to evaluate lymph node status and provide a systemic survey. Lymph nodes with short axes of > 1.0 cm on chest CT that showed fluorodeoxyglucose uptake on positron emission tomography/CT were clinically suspected to be metastatic. Endobronchial ultrasonography-guided transbronchial needle aspiration was performed for patients with suspicious hilar and mediastinal lymph nodes. Since the clinical and pathological stages were determined according to the 6th and 7th Editions of the TNM Classification in the initial period of this study, those cases were restaged according to the TNM 8th Edition for the present study. Postoperative complication severity was graded according to the Clavien–Dindo classification system [11].

### Definition of segmentectomy

Segmentectomy was classified into two categories: simple and complex. Simple segmentectomy includes resection of right 6th segment, basal (7th, 8th, 9th and 10th) segment, left 6th segment, left upper division segment, left lingular segment, and left basal (8th, 9th and 10th) segment. Complex segmentectomy includes any segmentectomy other than simple segmentectomy and those with subsegmentectomy.

### Surgical procedure

All the patients underwent standard general anesthesia with single-lung ventilation using a double-lumen endotracheal tube and were placed in the lateral decubitus position. Simple segmentectomy was performed via VATS during the period January 2011 to March 2020. Complex segmentectomy was performed via open thoracotomy from January 2011 to December 2017 and via VATS from January 2018 to March 2020. Open thoracotomy was defined as an approach using rib spreaders for 8- to 15-cm access and intercostal widening. Posterolateral or anterolateral thoracotomy was selected depending on the target segments. VATS segmentectomy was performed through 2- to 3-cm access incision and three thoracostomy ports completely under thoracoscopic visualization and using previously described techniques [12]. In open segmentectomy, the lung parenchyma to be resected was inflated to confirm the inflation-deflation lines after the pulmonary segmental vessels and bronchi were divided, after which the intersegmental planes were formed along the lines using an electrocautery scalpel and endo-staplers. In VATS segmentectomy, the intersegmental lines were confirmed by fluorescent thoracoscopy after systemic administration of indocyanine green (ICG), and intersegmental planes were formed only by endo-staplers. Open thoracotomy and VATS were defined as the conventional (CONV) group. From April 2020, both simple and complex segmentectomies were performed via RATS. In our institution, platforms used for RATS were the da Vinci Surgical System (DVSS) (Intuitive Surgical, Sunnyvale, CA. USA) second- and third-generation systems (da Vinci S, and Si, respectively) from January 2011 to December 2018, and DVSS fourth-generation systems (da Vinci X, and Xi, respectively) after January 2019. RATS was performed using previously described settings and techniques [13, 14]. In RATS segmentectomy, a vessel-sealing system (e.g., da Vinci Vessel Sealer Extend) was occasionally used to seal and cut thick tissues and small blood vessel branches. Da Vinci staplers (e.g., EndoWrist Staplers and SureForm) were used to staple pulmonary segmental arteries, veins and bronchi, and to divide the lung parenchyma to form the intersegmental plane. Intraoperative navigation of the anatomy was performed using the TilePro function to project the 3D-constructed CT on the screen to the surgeon console. To delineate intersegmental lines, systemic administration of ICG after dividing the pulmonary segmental arteries, and Firefly mode, which is an integrated fluorescence capability that uses near-infrared technology, was used. Mediastinal lymph node dissection was not mandatory for segmentectomy during the period of this study. The interlobar (#11), lobar (#12), and segmental (#13) lymph nodes were dissected separately; however, they were counted in the total because of their unclear dividing line.

### Statistical analysis

Before PSM, the Mann–Whitney *U* test for continuous covariates and Fisher’s exact test or the χ^2^ test was used for categorical covariates when comparing the two groups. Propensity scores were calculated by logistic regression modeling, including the clinical variables that might be considered as determinant factors in selecting the surgical approach; age, gender, smoking status, serum carcinoembryonic antigen level, percent vital capacity, forced expiratory volume in 1 s, C/T ratio, clinical stage, tumor laterality, and type of segmentectomy. We matched propensity scores one-to-one with the use of nearest-neighbor matching methods without replacement using a 0.1 caliper width. After matching procedure, 63 patients were selected for each group (CONV group and RATS group) for the analysis. After matching, the Wilcoxon signed-rank test for continuous covariates and the Mantel–Haenszel χ^2^ test for categorial covariates were used to compare those groups. Data were analyzed using SPSS statistical software version 22 (IBM, Armonk, NY, USA) and BellCurve for Excel (Social Survey Research Information, Tokyo, Japan). All statistical analyses were performed with the two-sided method, and a *p* value of < 0.05 (two-sided) was considered significant in all analyses.

## Results

### Clinicopathological characteristics

**Fig. 1:**
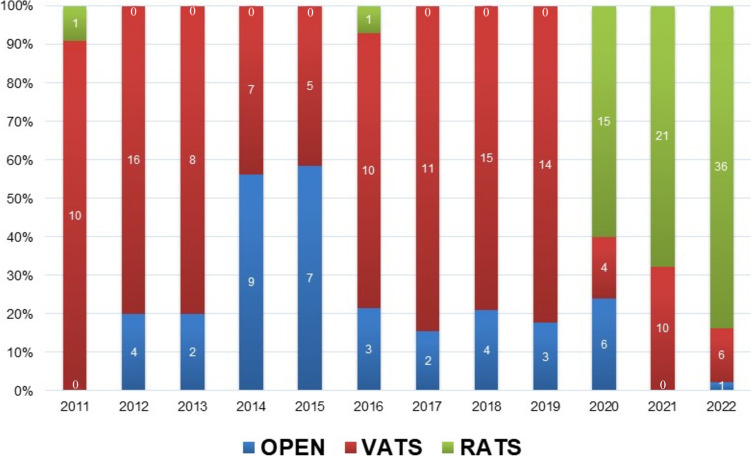
Trends of surgical approach of anatomical segmentectomy for primary lung cancer from 2011 to 2022. *OPEN* open thoracotomy, *VATS* video-assisted thoracoscopic surgery, *RATS* robot-assisted thoracoscopic surgery

Figure 1 shows the trends of our surgical approaches of anatomical segmentectomy for primary lung cancer. In April 2020, RATS segmentectomy started to be covered by the national health insurance system in Japan. The increased use of robotic approach was accompanied by a decreasing trend of conventional approaches from 100% in 2019 to 16% in 2022.

Table 1 shows the patients’ clinical characteristics before and after PSM. We included 231 patients (CONV, *n* = 157; RATS, *n* = 74) in this study. The patients comprised 127 men and 104 women, and their median age was 73 years (range 44–93 years). The study population comprised 122 ever smokers and 109 never smokers. The unmatched comparison showed significant differences in age (*p* = 0.028), smoking status (*p* = 0.025), and type of segmentectomy (*p* < 0.001), between the CONV and RATS groups. The data indicated that the RATS group contained older patients, never smokers, and patients who underwent complex segmentectomy. After PSM, 63 patients were selected for each group, and no differences in demographic data were found between the groups. Table 2 shows the patients’ pathological characteristics after PSM. There were no significant differences in pathologic characteristics between the CONV and RATS groups, other than vascular invasion. (*p* = 0.037).

**Table 1:** Clinical characteristics

	CONV		RATS		*p* value	CONV		RATS		*p* value
	*N* = 157	(% or range)	*N* = 74	(% or range)		*N* = 63	(% or range)	*N* = 63	(% or range)	
Age										
Median (IQR)	72 (13)	(44–93)	74.5 (9)	(56–86)	0.028	73 (13)	(49–92)	74 (9)	(56–86)	0.608
Gender										
Male	92	(59)	35	(47)	0.120	39	(62)	33	(52)	0.282
Female	65	(41)	39	(53)		24	(38)	30	(48)	
Smoking status										
Ever	91	(58)	31	(42)	0.025	34	(54)	30	(48)	0.478
Never	66	(42)	43	(58)		29	(46)	33	(52)	
Serum CEA level (ng/ml)										
Median (IQR)	3.1 (3.3)	(0.8–17.4)	2.6 (1.6)	(0.6–18.9)	0.071	3.1 (3.4)	(0.8–12.8)	2.6 (1.5)	(0.6–18.9)	0.207
%VC										
Median (IQR)	100 (22.4)	(48.4–157.1)	98.4 (24.5)	(66.4–126)	0.427	101.6 (25.1)	(67.1–135.9)	95.9 (22.5)	(66.4–126.0)	0.181
FEV_1.0%_										
Median (IQR)	74.2 (9)	(37.4–94.9)	75.6 (10.9)	(51.6–96.4)	0.334	74.4 (8.5)	(49.6–93.6)	75.3 (10.9)	(51.6–96.4)	0.457
C/T ratio										
Median (IQR)	0.72 (0.58)	(0–1)	0.65 (0.56)	(0–1)	0.725	0.72 (0.6)	(0–1)	0.71 (0.54)	(0–1)	0.766
c-Stage										
0	7	(4)	1	(1)	0.592	5	(8)	1	(2)	0.263
IA1	48	(31)	27	(36)		19	(30)	23	(37)	
IA2	56	(36)	28	(38)		20	(32)	22	(35)	
IA3	32	(20)	13	(18)		14	(22)	12	(19)	
IB	7	(4)	4	(5)		2	(3)	4	(6)	
II or more	7	(4)	1	(1)		3	(5)	1	(2)	
Tumor laterality										
Right	53	(34)	33	(45)	0.144	23	(37)	29	(46)	0.280
Left	104	(66)	41	(55)		40	(63)	34	(54)	
Type of segmentectomy										
Simple	128	(82)	35	(47)	< 0.001	40	(63)	35	(56)	0.366
Complex	29	(18)	39	(53)		23	(37)	28	(44)	

*CONV* conventional approach, *RATS* robot-assisted thoracoscopic surgery, *IQR* interquartile range, *CEA* carcinoembryonic antigen, *VC* vital capacity, *FEV* forced expiratory volume, *C/T ratio* consolidation-tumor ratio, *c-stage* clinical stage

**Table 2:** Pathological characteristics

	CONV		RATS		*p* value
	*N* = 63	(%)	*N* = 63	(%)	
Histology					
Adenocarcinoma	51	(81)	54	(86)	0.594
Squamous cell carcinoma	8	(13)	7	(11)	
Others	4	(6)	2	(3)	
p–T status					
Tis/T1mi/T1a	24	(38)	9	(14)	0.495
T1b	16	(25)	34	(54)	
T1c	7	(11)	5	(8)	
T2a	13	(21)	14	(22)	
T2b/3/4	3	(5)	1	(2)	
p-N status					
N0	60	(95)	59	(94)	0.698
N1/N2	3	(5)	4	(6)	
p-stage					
0	4	(6)	0	(0)	0.107
IA1	20	(32)	8	(13)	
IA2	16	(25)	35	(56)	
IA3	6	(10)	5	(8)	
IB	11	(17)	10	(16)	
II or more	6	(10)	5	(8)	
Pleural invasion (pl)					
Negative	52	(83)	57	(90)	0.194
Positive	11	(17)	6	(10)	
Lymphatic permeation (Ly)					
Negative	48	(76)	52	(83)	0.381
Positive	15	(24)	11	(17)	
Vascular invasion (V)					
Negative	43	(68)	53	(84)	0.037
Positive	20	(32)	10	(16)	

*CONV* conventional approach, *RATS* robot-assisted thoracoscopic surgery, *p–T* status pathological T status, *p-N status* pathological N status, *p-Stage* pathological stage

### Surgical outcomes

Surgical outcomes are summarized in Table 3, and Table 4 shows detailed data of postoperative complications. After PSM, there were significant differences between the CONV and RATS groups in total operative time (210 vs 154 min; *p* < 0.001), bleeding amounts (40 vs 10 mL; *p* < 0.001), and duration of chest drainage (2 vs 2 days; *p* = 0.041).

**Table 3:** Surgical outcomes

	CONV		RATS		*p *value
	*N* = 63	(% or range)	*N* = 63	(% or range)	
Total operative time					
Median (IQR)	210 (83.5)	(102–397)	154 (75.5)	(98–302)	< 0.001
Robotic console time					
Median (IQR)	–	–	111 (62)	(62–247)	–
Bleeding amount (mL)					
Median (IQR)	40 (75)	(5–345)	10 (5)	(5–110)	< 0.001
Duration of chest drain (days)					
Median (IQR)	2 (0)	(1–8)	2 (1)	(1–15)	0.041
Total postoperative complications					
No	52	(83)	55	(87)	0.457
Yes	11	(17)	8	(13)	
Prolonged air leak (CD ≥ G2)					
No	61	(97)	60	(95)	0.649
Yes	2	(3)	3	(5)	
Postoperative pneumonitis (CD ≥ G2)					
No	58	(92)	63	(100)	0.023
Yes	5	(8)	0	(0)	
Dissected hilar lymph nodes counts					
Median (IQR)	4 (6)	(0–17)	6 (6)	(0–20)	0.202

*CONV* conventional approach, *RATS* robot-assisted thoracoscopic surgery, *IQR* interquartile range, *CD* Clavien–Dindo

**Table 4:** Postoperative complications

		CONV (*N* = 63)	RATS (*N* = 63)
Postoperative complications (CD ≥ G2)			
Prolonged air leak		2 (3)	3 (5)
Bacterial pneumonia		4 (6)	0 (0)
Interstitial pneumonia		1 (1.6)	0 (0)
Empyema		1 (1.6)	0 (0)
Chylothorax		0 (0)	1 (1.6)
Atrial fibrillation		1 (1.6)	1 (1.6)
Recurrent laryngeal nerve palsy		1 (1.6)	1 (1.6)
Renal dysfunction		1 (1.6)	1 (1.6)
Cerebral infarction		0 (0)	1 (1.6)

*CONV* conventional approach, *RATS* robot-assisted thoracoscopic surgery, *CD* Clavien–Dindo

The two groups did not significantly differ in prevalence of total postoperative complications (*p* = 0.457), and prolonged air leak (*p* = 0.649); however, the incidence of postoperative pneumonia was significantly lower than in the RATS group (*p* = 0.023). The number of dissected hilar lymph nodes did not differ significantly between the groups (*p* = 0.202).

## Discussion

In this retrospective study, we reviewed the records of patients with primary lung cancer who underwent segmentectomy via RATS or conventional approaches, including open thoracotomy and VATS, and compared their clinicopathological characteristics and surgical outcomes after PSM. We found that RATS had significantly better surgical outcomes than the conventional approach, and the incidence of postoperative pneumonia was significantly lower in the RATS group.

Recent pivotal clinical trials have increased the importance of segmentectomy for small-sized, peripheral NSCLC [2–4]. Among the trials, JCOG0802/WJOG4607L is particularly valuable in that it demonstrated the superiority of segmentectomy over lobectomy regarding overall survival for early-stage NSCLC. Although there have been recently published interpretations of the results of these trials as well as editorials on the significance of segmentectomy for early-stage NSCLC and the debate over lobectomy versus segmentectomy [15–17], we are undoubtedly now moving from the era of lobectomy, which was considered the standard procedure for a long time based on the results of only one clinical trial [1], to a new era of the segmentectomy in terms of surgical procedure for small-sized, early-stage NSCLC.

In general, segmentectomy requires meticulous and careful handling of peripheral branches of pulmonary vessels and bronchus, which are smaller than those of lobectomies. In recent years, RATS has been considered useful for segmentectomy because the use of articulated robotic instruments in the thoracic cavity and 3D thoracoscopic view allows for more elaborate and complicated movements. When performing segmentectomy, the vascular and bronchial sheath must be more accurately grasped and more multi-directionally dissected in the proper layer of vessels. RATS has a great advantage in performing these manipulations, which is probably the main reason for the wider acceptance of RATS for segmentectomy. It is preferable in segmentectomy to perform the dissection of segmental vessels and bronchi while intraoperatively confirming the anatomy with 3D-constructed CT images in order to prevent misidentification of these branches. The TilePro mode in the DVSS, which is the function of direct visualization in a picture-on-picture fashion on the console screen, allow the thoracic surgeon to perform segmentectomy while confirming accurate anatomy of pulmonary vessels and bronchi [18]. This is an indispensable function for performing more complex segmentectomy properly and safely. Furthermore, intravascular administration of ICG has recently been introduced to delineate intersegmental lines, and the Firefly mode as a fluorescent endoscopy to distinguish the presence or absence of this blood flow is also a very useful function in RATS segmentectomy [19, 20]. If accurate intersegmental lines are able to be drawn using this function, stapling of intersegmental planes can be performed with sufficient surgical margin, which is of paramount importance in segmentectomy. In our study, the RATS group had favorable perioperative outcomes such as shorter operative time, less blood loss, and shorter duration of chest drainage, possibly due to the successful use of these unique functions of RATS.

Although there are some previous studies that compared the surgical outcomes of segmentectomy between RATS and other approaches, their results are not consistent [5–9]. With regard to operative time, some studies showed that RATS had a significantly longer duration than other approaches, while others showed that it was shorter. In our study, total operative time was significantly shorter in the RATS group than in the conventional-approach group. We offer two possible reasons for this result. First, we had more experience with RATS lobectomy and had mastered robotic surgical techniques because it began to be covered by national health insurance in our country 2 years earlier than RATS segmentectomy. Second, the advantages of innovative robotic technology have given impetus to the surgical techniques employed in segmentectomy. In particular, the “SmartFire technology” of SureForm allows us to perform the intersegmental formation in ideal directions, which not only shortens the operative time but also contributes to ensuring adequate surgical margins and preventing prolonged air leaks. Zhang et al. reported that experience with at least 40 cases was needed to pass the learning curve of RATS segmentectomies for small pulmonary lesions [21]; however, it may not take so much experience to master RATS segmentectomy if we can optimize the use of robotic technology such as aforementioned TilePro and Firefly mode with ICG administration, and stapling with robotic staplers.

In the current study, there was no significant difference in the incidence of all complications; however, the incidence of postoperative pneumonia was significantly lower in the RATS group than in the conventional-approach group. A previous study by Pan et al. compared the perioperative outcomes among RATS, VATS, and open lobectomy for NSCLC patients aged ≥ 75 years, in which they demonstrated that RATS led to the lowest incidence of postoperative complications, especially pneumonia, as well as shorter operative duration and less blood loss [22]. They speculated that these results may be due to mitigation of the impact of mechanical ventilation and anesthesia and altered internal environments for patients. Dexter et al. also revealed that increased odds of pneumonia for lobectomy were associated with longer procedure duration in their analysis using the Society of Thoracic Surgeons Database [23]. We believe that the results of these studies also apply to segmentectomy. In other words, the lower incidence of postoperative pneumonia in the RATS group in our study may have been due in part to favorable surgical outcomes including shorter operative time and less blood loss. Whether this point is correct needs to be clarified by a larger scale database analysis.

### Limitations

There are several limitations of this study, the first of which is its retrospective nature. Although selection biases were minimized by applying PSM with two evenly matched and comparable groups, uncontrolled selection bias might still exist because this study was not prospectively randomized and controlled. Second, the sample size was too small for us to reach a definitive conclusion because this was a single-institution study. Third, there was an evident “surgeon bias” in this study because most RATS segmentectomy procedures were performed chiefly by one three console surgeons, whereas open surgery and VATS were performed by several thoracic surgeons. Finally, we did not analyze the long-term survival of segmentectomy by RATS and other approaches. The overall survival advantage of segmentectomy for small-sized NSCLC was demonstrated in the JCOG0802 trial [2], and it is basically not acceptable to permit differences in long-term survival according to surgical approach, so we need to confirm that RATS segmentectomy is not inferior to other surgical approaches in terms of long-term survival.

## Conclusion

Our study demonstrated that RATS segmentectomy had better surgical outcomes, including shorter operative time, less blood loss, shorter duration of chest drainage, and decreased incidence of postoperative pneumonia, than open thoracotomy or VATS. Further studies on oncological long-term outcomes of RATS segmentectomy and comparative analysis of operative costs are needed in the future.

### Supplementary Information

Below is the link to the electronic supplementary material.

Supplementary file1 (DOCX 32 KB)

## Data Availability

The data underlying this article will be shared on reasonable request to the corresponding author.

## Abbreviations

CT: Computed tomography
DVSS: Da Vinci Surgical System
ICG: Indocyanine green
JCOG: Japan Clinical Oncology Group
NSCLC: Non-small cell lung cancer
PSM: Propensity-score matching
RATS: Robot-assisted thoracoscopic surgery
VATS: Video-assisted thoracoscopic surgery
