# Supplementary material for: A comparative study of robot-assisted thoracoscopic surgery and conventional approaches for short-term outcomes of anatomical segmentectomy
Source: Gen Thorac Cardiovasc Surg. 2023 Nov 7;72(5):338–45. doi: 10.1007/s11748-023-01983-y (PMC11018688; doi:10.1007/s11748-023-01983-y)
Supplement: Supplementary file 1 — Supplementary file1 (DOCX 32 KB) [file 11748_2023_1983_MOESM1_ESM.docx]

**Supplemental table 1**

*Pathological characteristics before propensity score matching*

|  |  | CONV | | RATS | | *p*-value |
| --- | --- | --- | --- | --- | --- | --- |
|  |  | N = 157 | (%) | N = 74 | (%) |  |
| Histology | |  |  |  |  |  |
| Adenocarcinoma | | 133 | (85) | 65 | (88) | 0.761 |
| Squamous cell carcinoma | | 17 | (11) | 7 | (9) |  |
| Others | | 7 | (4) | 2 | (3) |  |
| p-T status | |  |  |  |  |  |
| Tis/T1mi/T1a | | 51 | (32) | 12 | (16) | 0.080 |
| T1b | | 58 | (37) | 38 | (51) |  |
| T1c | | 18 | (11) | 9 | (12) |  |
| T2a | | 25 | (16) | 14 | (19) |  |
| T2b/3/4 | | 5 | (3) | 1 | (1) |  |
| p-N status | |  |  |  |  |  |
| N0 | | 145 | (92) | 70 | (95) | 0.532 |
| N1/N2 | | 12 | (8) | 4 | (5) |  |
| p-stage | |  |  |  |  |  |
| 0 | | 5 | (3) | 0 | (0) | 0.038 |
| IA1 | | 45 | (29) | 11 | (15) |  |
| IA2 | | 55 | (35) | 40 | (54) |  |
| IA3 | | 17 | (11) | 8 | (11) |  |
| IB | | 18 | (11) | 10 | (14) |  |
| II or more | | 17 | (11) | 5 | (7) |  |
| Pleural invasion (pl) | |  |  |  |  |  |
|  | Negative | 133 | (85) | 68 | (92) | 0.130 |
|  | Positive | 24 | (15) | 6 | (8) |  |
| Lymphatic permeation (Ly) | |  |  |  |  |  |
|  | Negative | 128 | (82) | 63 | (85) | 0.499 |
|  | Positive | 29 | (18) | 11 | (15) |  |
| Vascular invasion (V) | |  |  |  |  |  |
|  | Negative | 130 | (83) | 64 | (86) | 0.476 |
|  | Positive | 27 | (17) | 10 | (14) |  |

CONV, conventional approach; RATS, robot-assisted thoracoscopic surgery; p-T status, pathological T status; p-N status, pathological N status; p-Stage, pathological stage

**Supplemental table 2**

*Surgical outcomes before propensity score matching*

|  |  | CONV | | RATS | | *p*-value |
| --- | --- | --- | --- | --- | --- | --- |
|  |  | N = 157 | (% or range) | N = 74 | (% or range) |  |
| Total operative time | |  |  |  |  |  |
| Median (IQR) | | 183 (70) | (70 - 397) | 155 (80) | (98 - 302) | 0.036 |
| Robotic console time | |  |  |  |  |  |
| Median (IQR) | | ― | ― | 111 (62) | (59 – 247) | ― |
| Bleeding amount (mL) | |  |  |  |  |  |
| Median (IQR) | | 10 (45) | (3 - 1340) | 5 (5) | (5 -110) | 0.010 |
| Duration of chest drain (days) | |  |  |  |  |  |
| Median (IQR) | | 2 (0) | (1 - 11) | 2 (1) | (1 - 15) | < 0.001 |
| Total postoperative complications | |  |  |  |  |  |
|  | No | 127 | (81) | 65 | (88) | 0.189 |
|  | Yes | 30 | (19) | 9 | (12) |  |
| Prolonged air leak (CD≥G2) | |  |  |  |  |  |
|  | No | 152 | (97) | 71 | (96) | 0.736 |
|  | Yes | 5 | (3) | 3 | (4) |  |
| Postoperative pneumonitis (CD≥G2) | |  |  |  |  |  |
|  | No | 149 | (95) | 74 | (100) | 0.048 |
|  | Yes | 8 | (5) | 0 | (0) |  |
| Dissected hilar lymph nodes counts | |  |  |  |  |  |
| Median (IQR) | | 4 (4) | (0 – 18) | 4 (4) | (0 – 20) | 0.255 |

CONV, conventional approach; RATS, robot-assisted thoracoscopic surgery; IQR, interquartile range; CD, Clavien-Dindo

**Supplemental table 3**

*Postoperative complications before propensity score matching*

|  | |  |  | CONV (N = 157) | | RATS (N = 74) | |
| --- | --- | --- | --- | --- | --- | --- | --- |
| Postoperative complications (CD≥G2) | | | |  |  |  |  |
|  | Prolonged air leak | | | 5 (3) | | 3 (4) | |
|  | Bacterial pneumonia | | | 8 (5) | | 0 (0) | |
|  | Interstitial pneumonia | | | 1 (0.6) | | 0 (0) | |
|  | Empyema | | | 1 (0.6) | | 0 (0) | |
|  | Chylothorax | | | 0 (0) | | 1 (1.3) | |
|  | Atrial fibrillation | | | 4 (2.5) | | 1 (1.3) | |
|  | Recurrent laryngeal nerve palsy | | | 1 (0.6) | | 1 (1.3) | |
|  | Renal dysfunction | | | 1 (0.6) | | 1 (1.3) | |
|  | Cerebral infarction | | | 0 (0) | | 1 (1.3) | |
|  | Hyperkalemia | | | 1 (0.6) | | 0 (0) | |
|  | Gastroduodenal ulcer | | | 2 (1.3) | | 0 (0) | |
|  | Others | | | 2 (1.3) | | 0 (0) | |

CONV, conventional approach; RATS, robot-assisted thoracoscopic surgery; CD, Clavien-Dindo
